# BPNN-Based Behavioral Modeling of the S-Parameter Variation Characteristics of PAs with Frequency at Different Temperatures

**DOI:** 10.3390/mi13111831

**Published:** 2022-10-26

**Authors:** Zhao He, Shaohua Zhou

**Affiliations:** 1 National Institute of Metrology, Beijing 100029, China; 2 School of Microelectronics, Tianjin University, Tianjin 300072, China

**Keywords:** power amplifier, temperature, BPNN, S-parameter, model

## Abstract

To address the issue of frequency nonlinearity modeling of RF PAs, which is rarely seen in the literature, a BPNN is applied to model the frequency nonlinearity of RF PAs in this paper. The BPNN is used to model the frequency nonlinearity of the RF PA, based on the actual measured S-parameter data at different ambient temperatures. The modeling results show that BPNN shows the advantage of a high accuracy in modeling the frequency nonlinearity of RF PAs. It is expected that a BPNN will also show the advantages of a high accuracy in the modeling process of other RF devices or circuits.

## 1. Introduction

A radio-frequency (RF) power amplifier (PA) is a key component of the wireless system, and its specification directly affects the system’s performance [1,2,3]. Of all of these key specifications, linearity and efficiency are the two most important, from a system application perspective [4]. However, due to the inherent nonlinear behavior of PAs, it is difficult to achieve both a high linearity and a high efficiency [5]. Therefore, the power amplifier must be linearized to maintain a good linearity while improving efficiency [6]. The linearization of PAs is to model the behavior of PAs and then compensate for the nonlinear characteristics of the PAs [6].

The current PA behavioral modeling focuses on modeling the PA’s amplitude nonlinearity (the relationship between input and output power), i.e., describing the specific nonlinear characteristics of its amplitude for specific measured input and output data. However, as the communication rate demand increases, the operating bandwidth under the linear operation becomes wider [7,8,9], resulting in an increasing frequency nonlinear effect. Therefore, in addition to the traditional amplitude nonlinearity, the frequency nonlinear effect affecting the linear operating condition of the wireless communication systems must become an urgent factor to be considered. Furthermore, the characteristics of the triode itself are also a nonlinear function of frequency, making the S-parameters of the PA in the linear operating condition strongly correlated with frequency. Therefore, to know the relationship between the nonlinear characteristics and frequency over the entire bandwidth, it is necessary to model the S-parameters of the PA with frequency in the same way as the amplitude nonlinearity is modeled for the PA, to provide a basis for compensation of the nonlinear characteristics with frequency.

Modeling the amplitude nonlinearity of a power amplifier means choosing some suitable function or way to describe the relationship between the input and output power of a RF power amplifier [10]. Therefore, for the frequency domain modeling of the S-parameters, it is likewise a matter of choosing some appropriate function or way to describe the relationship between the S-parameters and the frequency of the RF power amplifier.

A number of methods for describing the PA behavior have been reported in the literature. For example, in 2005, D. Ronnow et al. of the University of Gävle, used the radial basis function neural networks (RBFNNs) for modeling the PA’s nonlinear behavior [11]. In 2006, Thomas J. Brazil et al. of University College Dublin, Ireland, applied a simplified Volterra model to model the PA’s behavior [12]. In 2009, Slim Boumaiza et al. applied a novel Hammerstein model to model the PA’s behavior [13]. In 2011, Farouk Mkadem et al. proposed a novel two-hidden-layer artificial neural network (2HLANN) model and used it for modeling the PA’s behavior [14]. A modified canonical piecewise-linear (CPWL) function was proposed by Anding Zhu et al., in 2015, and used it to model the behavior of PAs [15]. In 2016, Slim Boumaiza et al. from the University of Waterloo, modeled the behavior of PAs using a generalized memory polynomial (GMP) model [16]. In 2019, Jialin Cai et al. modeled the behavior of PAs using a time-delayed support vector regression (SVR) method [17]. In 2020, Fadhel M. Ghannouchi et al. from the University of Calgary, used deep neural networks (DNNs) to model PAs’ behavior [18]. In 2020, Gaoming Xu et al. from Ningbo University, China, proposed a behavioral model consisting of Chebyshev polynomials (CP) and a long short-term memory (LSTM) network, i.e., CP-LSTM, for the PA’s behavioral modeling [19]. In 2022, we modeled the behavior of the PA’s temperature characteristics, using a support vector machine (SVM) and extreme learning machine (ELM) [20,21], respectively.

All of these studies above model the amplitude nonlinearity of a PA at room temperature and lack the description of the nonlinear characteristics of the PA as a function of frequency. By analyzing the above literature, various functions or ways are quickly used to describe the amplitude nonlinearity of a PA, which indicates that no function or method can perfectly describe the nonlinear characteristics of a PA. Therefore, whenever new functions or ways appear, they are quickly used to describe the PA nonlinear properties. The same applies to describing PAs’ nonlinear characteristics as a frequency function.

A back propagation neural network (BPNN), one of the most widely known neural networks with a high learning accuracy [22], has been commonly used in fields, such as microwave device modeling and antenna design [23,24], and is expected to show its superiority in behavioral modeling describing the S-parameters of PAs at room temperature, as a function of frequency.

When the ambient temperature changes, the S-parameters and the relationship between the S-parameters and frequency also change (degradation of the S-parameters concerning the typical S-parameters with frequency, at room temperature). Therefore, it is necessary to model not only the S-parameters of the PA, at room temperature in the frequency domain, but also the measured data of the S-parameters of the PA at different temperatures, concerning frequency, to provide a basis for the compensation of the S-parameter variation characteristics, at different temperatures relating to frequency. This ensures that the PA can always maintain consistent (or acceptable) characteristics over the entire bandwidth at different temperatures. Unfortunately, modeling the S-parameter versus frequency at room or non-room temperature, is rarely seen in the current literature.

This paper uses a BPNN to model the behavior of a 0.3–1.1 GHz CMOS wideband PA, in the frequency domain, with measured S-parameters at different temperatures. This PA was implemented using a 0.18μm CMOS process. The modeling results are compared with a SVM. The experimental results show that the BPNN can effectively characterize the S-parameter variation of the PA with frequency at different temperatures with a high accuracy.

## 2. Modeling Process Based on a BPNN

### 2.1. The Theory of a BPNN

**Figure 1:**
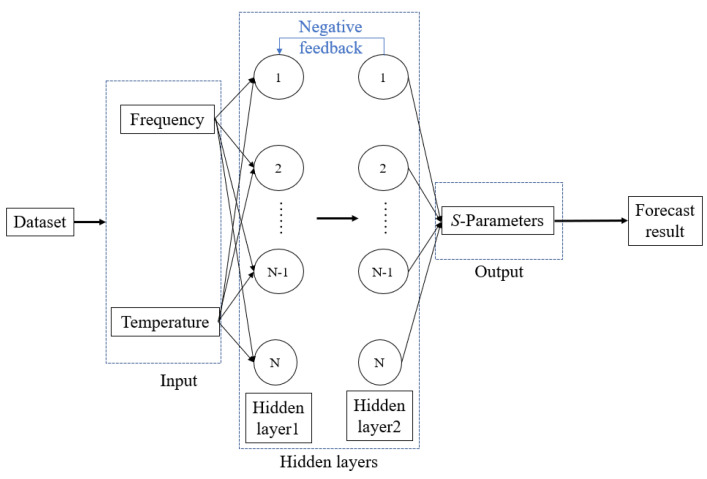
Schematic diagram of the structure of the BPNN.

BP (back propagation) neural network, a concept proposed by scientists led by Rumelhart and McClelland in 1986, is a multilayer feedforward neural network trained, according to the error back propagation algorithm and is one of the most widely used neural network models [25]. BP neural networks generally contain an input layer, an output layer, and an implicit layer, where the input layer uses known data, and the output layer is used to output the model predictions [25,26]. The implicit layer can contain a single layer or multiple layers, and the most basic BPNN generally has only one hidden layer [26]. In this paper, for example, a two-layer hidden layer BPNN structure is used, which is schematically as shown in Figure 1.

From this, the learning process of the BPNN can be divided into two parts: the forward transmission of information and the backward propagation of errors [25]. The forward transmission of information is to pass the information from the input layer to the output layer through the processing of the hidden layer. If the output does not reach the predetermined target, the change in the error of the output layer is requested and then back propagated by the network structure, to reverse the error [25,26]. The error signal is propagated backward through the network structure, according to the original connection path, and the weights and thresholds of the neurons in each layer are changed to make them meet the predefined conditions [25,26]. The network structure then propagates the error signal backward according to the initial connection path and then changes the weights and thresholds of the neurons in each layer to make them satisfy the pre-condition [25].

The model used in this paper is a two-layer BPNN model, which has input nodes *P_j_* (*j* = 1, 2, …r), and the hidden layer nodes are *a*_1*i*_ (*i* = 1, 2, …s_1_), the activation function is *F*_1_, the output node is *a*_2*k*_ (*k* = 1, 2, …s_2_), the activation function is *F*_2_, the weights between the input and the hidden layers are *w*_1*ij*_, the weights between the hidden and output layers are *w*_2*ki*_, the output is A, and the target vector of the BPNN model is *T* [25]. Based on the above discussion, the specific calculation procedure of the BPNN model coefficients is as follows.

The output of the *i*th node of the hidden layer is [25]

(1) $$a_{1i}=f_{1}(\sum_{j=1}^{r}w_{1ij}P_{j}+b_{1i})$$

The output of the *k*th node of the output layer is [25]

(2) $$a_{2k}=f_{2}(\sum_{i=1}^{s_{1}}w_{2ki}a_{1i}+b_{2i})$$

The error can be expressed as follows [25].

(3) $$E=\frac{1}{2}{\left(\sum_{k=1}^{s_{2}}t_{k}-a_{2k}\right)}^{2}$$

The back-propagation of the error can then be derived by the gradient descent method, where the change in the output layer weights and the change in the output layer threshold are [25]

(4) $$\begin{array}{l} \Delta w_{2ki}=-\eta \frac{\partial E}{\partial w_{2ki}}=\eta \times \delta_{ki}\times a_{1i}\\ \delta_{ki}=e_{k}\times f_{2}^{\prime }\\ e_{k}=t_{k}-a_{2k} \end{array}$$

(5) $$\Delta b_{2ki}=-\eta \frac{\partial E}{\partial b_{2ki}}=\eta \times \delta_{ki}$$

Similarly, it can be deduced that the change in the hidden layer weight and the change in the hidden layer threshold, are as follows [25].

(6) $$\begin{array}{l} \Delta w_{1ij}=-\eta \frac{\partial E}{\partial w_{1ij}}=\eta \times \delta_{ij}\times P_{j}\\ \delta_{ij}=e_{i}\times f_{1}^{\prime }\\ e_{i}=\sum_{k=1}^{s_{2}}\delta_{ki}w_{2ki} \end{array}$$

(7) $$\Delta b_{1i}=\eta \times \delta_{ij}$$

According to the above formula, since the hidden function layer has no target vector, the product of the error of the output layer and the derivative of the activation function of the output layer can be taken as the target vector of the hidden function layer [25,26]. Then the change of the weights of the implicit function layer is derived by the backward transfer of the error, and finally, the change of the weights of the previous layer is derived, according to the error of the implicit function, until the end of the derivation to the first layer [25,26].

### 2.2. Training Process of the BPNN Model

**Figure 2:**
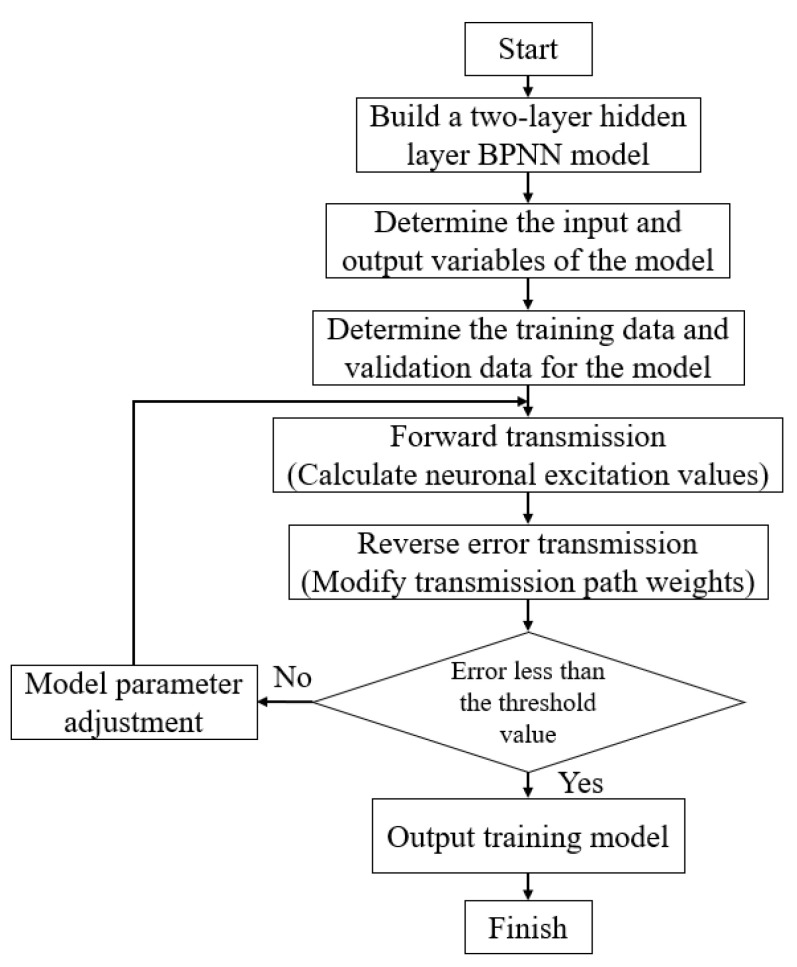
Modeling process based on the BPNN.

Figure 2 shows the modeling flow of the relationship between the S-parameters of 0.3–1.1 GHz CMOS PA and the frequency at different temperatures.

This paper uses the neural network toolbox in MATLAB to implement the model’s training and validation. First, it obtains the S-parameter data required for modeling, based on the S-parameter file (with the .s2p extension) obtained from the vector network analyzer measurements, and then imports it into the advanced design system (ADS). The training process, based on the BPNN model, consists of the following steps.

Step 1: Establishing a BPNN model with a two-layer hidden layer, according to Figure 1.

Step 2: Determine the input and output variables of the model, the input variables, which are frequency and temperature, and the output variables of the model are the S-parameters.

Step 3: Determine the training and validation data of the model, where 50% of the measured data of the 0.3–1.1 GHz CMOS PA is the training data, and the other 50% of the measured data is the validation data of the model.

Step 4: Forward transmission, calculating the excitation values of the neurons in [27].

Step 5: Reverse error transmission, modifying the weights of the transmission paths in [27].

Step 6: Compare the model’s error with the threshold value. If the error is less than the threshold value, then the model training is completed. However, if the error is greater than the threshold, the model training needs to be repeated. The training process can be carried out by adjusting the model parameters, such as the number of hidden layers, the number of hidden layer neurons, and the learning rate, until the model error is less than the threshold [26,28].

Once the model is trained, it needs to be validated. Then, the trained model is used to predict the values of the S-parameters of the untrained points. The predicted results are then compared with the validation data.

(1) If the validation result is greater than the threshold, the model is over-learning and needs to be retrained.
(2) If the validation result is less than the threshold, the model is in a good learning state and can be used.

## 3. Modeling Results

### 3.1. The Result of S_11_

**Figure 3:**
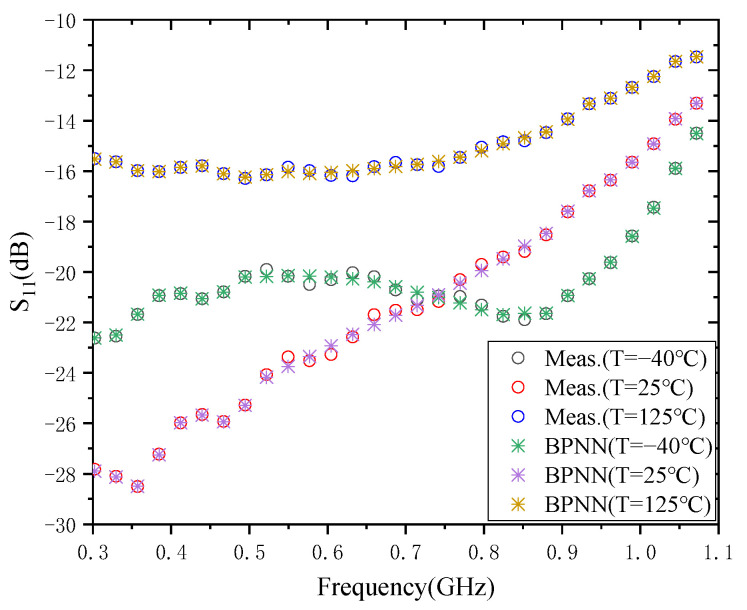
Modeling results of S_11_.

The modeling result of S_11_ for 0.3–1.1 GHz CMOS PA, is given in Figure 3.

**Figure 4:**
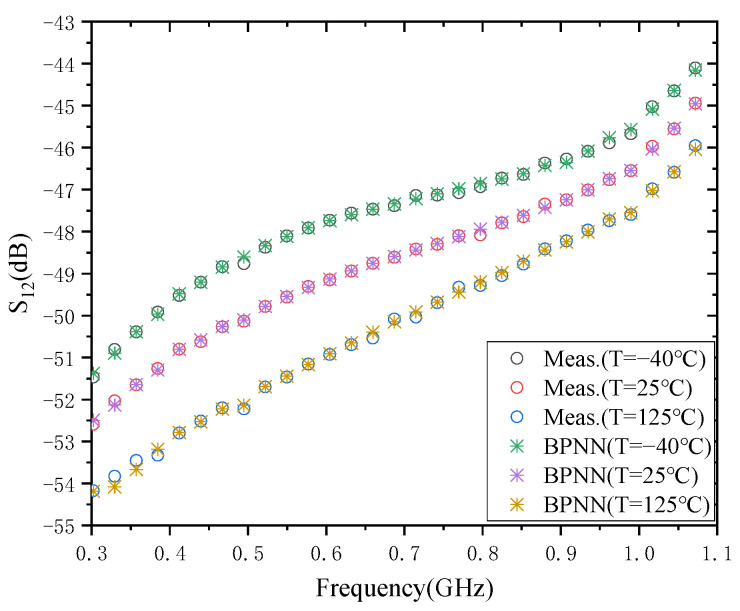
Modeling results of S_12_.

**Figure 5:**
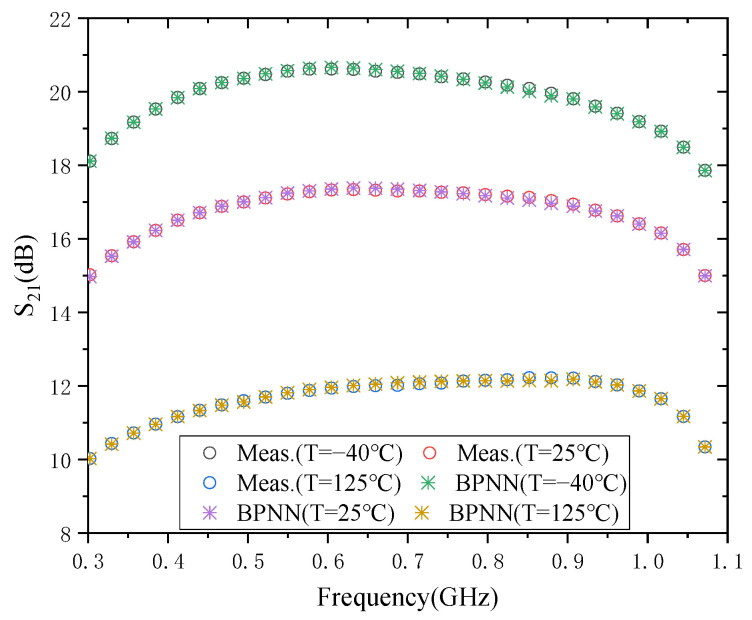
Modeling results of S_21_.

**Figure 6:**
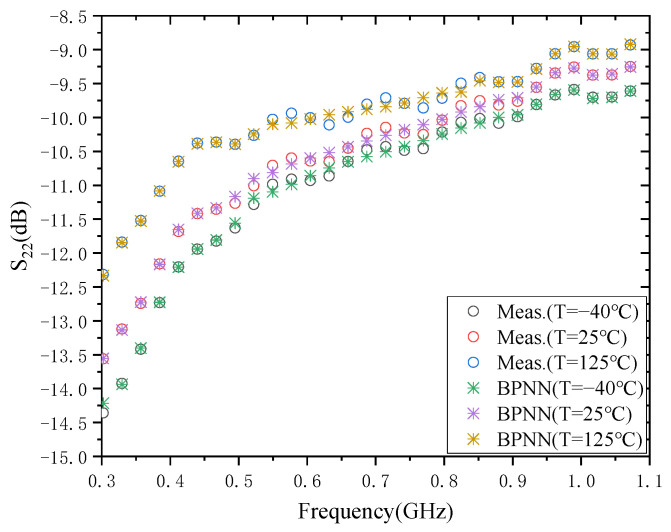
Modeling results of S_22_.

The “Meas.” in Figure 3, Figure 4, Figure 5 and Figure 6 represent the results of the experimental measurements. The instruments and equipment, such as a vector network analyzer, an environmental test chamber (SC^3^ 1000 MHG), and a DC power supply were used in the measurement process.

For the modeling results of S_11_, the training time required for the BPNN model is 4.143 × 10^3^ ms, 4.247 × 10^3^ ms, and 4.357 × 10^3^ ms for the three temperatures of −40 °C, 25 °C, and 125 °C, respectively. The test errors of the models were 1.7765 × 10^−2^, 3.2386 × 10^−2^, and 9.3347 × 10^−3^, respectively. The S_11_ has the smallest test error at 125 °C and the largest at a temperature of 25 °C. One possible reason for this phenomenon is the magnitude of the magnitude difference of S_11_ over the entire frequency range. The larger the magnitude of S_11_, the larger the measurement error of the model.

### 3.2. The Result of S_12_

The modeling result of S_12_ is shown in Figure 4. The training time required for the BPNN-based model is about 400 ms at the three temperatures of −40 °C, 25 °C, and 125 °C. The test errors of S_12_ at the three temperatures were 4.2475 × 10^−3^, 3.3293 × 10^−3^, and 7.3449 × 10^−3^, respectively. The test error is the largest at 125 °C and the smallest at 25 °C. A possible reason for this phenomenon is also related to the amplitude difference of S_12_ over the whole frequency range. The reason why the accuracy of the modeling results of S_12_ at 125 °C is lower than the accuracy of the model at 25 °C, is the same as that of S_11_. As can be seen from the figure, the amplitude difference of S_12_ is the largest at 125 °C, and therefore its model has the largest test error.

### 3.3. The Result of S_21_

The modeling result of the BPNN of S_21_ is given in Figure 5. From the time perspective, the training time required for the model is 0.555 × 10^3^ ms, 0.547 × 10^3^ ms, and 0.541 × 10^3^ ms, at the three temperatures of −40 °C, 25 °C, and 125 °C, respectively. It means that the required training time of the model is around 500 ms at these three temperatures. The required training times for S_11_ and S_12_ are around 4000 ms and 400 ms, respectively. This is mainly due to the difference in the number of hidden layer neurons used in each specification during training.

From the perspective of model test errors, the model test errors at the three temperatures were 9.7461 × 10^−4^, 8.711 × 10^−4^, and 8.2218 × 10^−4^, respectively. It indicates that the test error accuracy of the S_21_ model can reach the order of 10^−4^ at the three temperatures of −40 °C, 25 °C, and 125 °C. S_11_ and S_12_ can reach 10^−2^ and 10^−3^ orders of magnitude, respectively. However, it should be noted that by increasing the number of hidden layers of the BPNN model, the model accuracy of S_11_, S_12,_ and S_21_ can be further improved, which of course, causes a further increase in the time required for the model training. Therefore, the two elements of time and accuracy need to be considered together in the modeling process.

### 3.4. The Result of S_22_

The modeling results of S_22_ at three temperatures, −40 °C, 25 °C, and 125 °C, are shown in Figure 6. The training times required for the model at these three temperatures are 1.479 × 10^3^ ms, 1.442 × 10^3^ ms, and 1.427 × 10^3^ ms, respectively. The test errors of the models were all in the order of 10^−3^, specifically 3.5154 × 10^−3^, 4.7687 × 10^−3^, and 3.7158 × 10^−3^. As with the model accuracy of S_11_, S_12,_ and S_21_, the model accuracy of S_22_ can also be further improved by increasing the number of hidden layers. However, only the corresponding model training time will be increased. It also shows that the BPNN model is effective for the prediction of the temperature characteristics of S_22_.

## 4. Discussion

The modeling results of S_11_, S_12_, S_21_, and S_22_ show that the BPNN model can be effectively used to describe the relationship between the S-parameter and frequency at different temperatures. If further improvement in the model’s accuracy is needed, it can be achieved by increasing the number of hidden layers of the model, although this causes an increase in the training time required for the model.

The literature [20] used the SVM to model the S-parameters of a 2.5–5.2 GHz CMOS PA, and the results show that the SVM can be used to model the S-parameters. Therefore, this paper has modeled the S-parameters of a 0.3–1.1 GHz CMOS PA, using a BPNN and SVM, respectively, and tried to compare these two models in terms of modeling speed and accuracy, and the results are shown in Table 1.

From a time perspective, the training time required for the BPNN model far exceeds the training time required for the SVM model. From the perspective of model accuracy, the model accuracy of the BPNN is much higher than the accuracy of the SVM model. That is, the prediction error of the BPNN model is much lower than that of the SVM model. This shows that the BPNN model is very effective in modeling the behavior of the frequency-dependent S-parameters of 0.3–1.1 GHz CMOS power amplifiers at different temperatures. It can also be seen that the model training time is shorter for S_12_ and S_21,_ than for S_11_ and S_22_, which is mainly due to the fact that fewer neurons are used in the model training process for S_12_ and S_21,_ than for S_11_ and S_22_. Therefore, if online training is required, the shorter the training time of the model, the less critical the training time of the model is, if it is trained offline.

It should also be noted that this paper adopts a two-layer hidden layer BPNN model structure, so the accuracy of the BPNN model can be further improved by increasing the number of hidden layers and adjusting the number of neurons in each hidden layer, but the corresponding model training time will also increase. Therefore, a compromise between the model accuracy and time is needed when applying the BPNN to model the behavior of the frequency-dependent S-parameters at different temperatures.

To sum up, from the time required for model training and accuracy, the SVM model and the BPNN model have their advantages and disadvantages, which also shows that no function or method can completely describe the nonlinear characteristics of the PA’s S parameter changing with frequency. Moreover, it can be imagined that the various functions or methods used to describe the nonlinear amplitude characteristics of the PA will also be quickly used to describe the nonlinear variation with frequency in the entire bandwidth of the various RF PAs.

## 5. Conclusions

Based on the analysis of the current state of research on the behavior modeling of RF PAs, little modeling of the frequency nonlinearity of RF PAs has been seen. Based on the measured S-parameter variation with the frequency of RF PAs, the BPNN is applied to model the frequency nonlinearity, and the obtained modeling results are analyzed and validated. The BPNN shows the advantage of a high accuracy in modeling the frequency nonlinearity of RF PAs. Therefore, it can be expected that the BPNN can be used, not only for modeling other RF devices or circuits, but also will show the same advantages of a high accuracy, as the modeling of frequency nonlinearity of RF PAs.

## Figures and Tables

**Table 1:** Comparison of modeling results of the BPNN and SVM.

Specification	Temperature	Training Time (ms)		Training Error (MSE)		Test Error (MSE)	
		BPNN	SVM [20]	BPNN	SVM [20]	BPNN	SVM [20]
S_11_	−40 °C	4.143 × 10^3^	27.5078	1.7912 × 10^−2^	6.5025 × 10^−2^	1.7765 × 10^−2^	6.3965 × 10^−2^
	25 °C	4.247 × 10^3^	27.2977	3.2763 × 10^−2^	1.3129 × 10^−1^	3.2386 × 10^−2^	1.3112 × 10^−1^
	125 °C	4.357 × 10^3^	27.0359	9.3889 × 10^−3^	2.2186 × 10^−2^	9.3347 × 10^−3^	2.1894 × 10^−2^
S_12_	−40 °C	0.412 × 10^3^	27.058	5.5512 × 10^−3^	4.1416 × 10^−1^	4.2475 × 10^−3^	4.1301 × 10^−1^
	25 °C	0.467 × 10^3^	27.4604	4.4496 × 10^−3^	3.3189 × 10^−1^	3.3293 × 10^−3^	3.3065 × 10^−1^
	125 °C	0.47 × 10^3^	27.3995	8.8606 × 10^−3^	1.4380	7.3449 × 10^−3^	1.4317
S_21_	−40 °C	0.555 × 10^3^	27.2545	9.7952 × 10^−4^	1.8441 × 10^−1^	9.7461 × 10^−4^	1.8134 × 10^−1^
	25 °C	0.547 × 10^3^	27.2932	9.0344 × 10^−4^	1.8858 × 10^−1^	8.711 × 10^−4^	1.8705 × 10^−1^
	125 °C	0.541 × 10^3^	26.9892	8.4794 × 10^−4^	3.3277 × 10^−1^	8.2218 × 10^−4^	3.2986 × 10^−1^
S_22_	−40 °C	1.479 × 10^3^	27.2581	3.9397 × 10^−3^	1.7729 × 10^−1^	3.5154 × 10^−3^	1.6534 × 10^−1^
	25 °C	1.442 × 10^3^	27.191	4.8002 × 10^−3^	1.8305 × 10^−1^	4.7687 × 10^−3^	1.5922 × 10^−1^
	125 °C	1.427 × 10^3^	27.3121	3.7454 × 10^−3^	1.9121 × 10^−1^	3.7158 × 10^−3^	1.8743 × 10^−1^

## Data Availability

Not applicable.
